# Association Between Paternal Age and Birth Weight in Preterm and Full-Term Birth: A Retrospective Study

**DOI:** 10.3389/fendo.2021.706369

**Published:** 2021-07-22

**Authors:** Yiting Mao, Chen Zhang, Yinyu Wang, Yicong Meng, Lei Chen, Cindy-Lee Dennis, Jianzhong Sheng, Yanting Wu, Hefeng Huang

**Affiliations:** ^1^ The International Peace Maternity and Child Health Hospital, School of Medicine, Shanghai Jiao Tong University, Shanghai, China; ^2^ Shanghai Key Laboratory of Embryo Original Disease, Shanghai, China; ^3^ Obstetrics and Gynecology Hospital of Fudan University, Shanghai, China; ^4^ Bloomberg Faculty of Nursing, University of Toronto, Toronto, ON, Canada; ^5^ Department of Pathology and Pathophysiology, School of Medicine, Zhejiang University, Hangzhou, China

**Keywords:** paternal age at birth, birth weight, large for gestational age, obesity, small for gestation age

## Abstract

**Purpose:**

While it is well documented that maternal adverse exposures contribute to a series defects on offspring health according to the Developmental Origins of Health and Disease (DOHaD) theory, paternal evidence is still insufficient. Advanced paternal age is associated with multiple metabolism and psychiatric disorders. Birth weight is the most direct marker to evaluate fetal growth. Therefore, we designed this study to explore the association between paternal age and birth weight among infants born at term and preterm (<37 weeks gestation).

**Methods:**

A large retrospective study was conducted using population-based hospital data from January 2015 to December 2019 that included 69,964 cases of singleton infant births with complete paternal age data. The primary outcome was infant birth weight stratified by sex and gestational age including small for gestational age (SGA, 10th percentile) and large for gestational age (LGA, 90th percentile). Birth weight percentiles by gestational age were based on those published in the INTERGROWTH-21st neonatal weight-for gestational-age standard. Logistic regression analysis and linear regression model were used to estimate the association between paternal age and infant birth weight.

**Results:**

Advanced paternal age was associated with a higher risk for a preterm birth [35–44 years: adjusted odds ratio (OR) = 1.13, 95%CI (1.03 to 1.24); >44 years: OR = 1.36, 95%CI (1.09 to 1.70)]. Paternal age exerted an opposite effect on birth weight with an increased risk of SGA among preterm infants (35–44years: OR = 1.85, 95%CI (1.18 to 2.89) and a decreased risk among term infant (35–44years: OR = 0.81, 95%CI (0.68 to 0.98); >44 years: OR = 0.50, 95%CI (0.26 to 0.94). U-shaped associations were found in that LGA risk among term infants was higher in both younger (<25 years) (OR = 1.32; 95%CI, 1.07 to 1.62) and older (35–44 years) (OR = 1.07; 95% CI, 1.01 to 1.14) fathers in comparison to those who were 25 to 34 years old at the time of delivery.

**Conclusions:**

Our study found advanced paternal age increased the risk of SGA among preterm infants and for LGA among term infants. These findings likely reflect a pathophysiology etiology and have important preconception care implications and suggest the need for antenatal monitoring.

## Introduction

While it is well documented that advanced age among women is an important risk factor for infertility, miscarriage, and offspring genetic defects (1), less is known about the effects of advanced age on reproductive impairment among men. Globally, paternal age at childbirth is steadily increasing. In 1993, the proportion of fathers aged 35 to 54 in the UK was 25%, and in 2003 this proportion rose to 40% (2). From 1972 to 2015, the National Vital Statistics System data has shown that the mean paternal age has increased from 27.4 to 30.9 years in US (3). In 2014, the Chinese government started to implement an exemption to the only-child policy allowing a second child which contributed to the phenomenon that many older couples deciding to have a second child. Researchers have shown that the risk increase in incontrovertible paternal age-related adverse conditions starts around 35 years old of paternal age (4). There is preliminary evidence to suggest that advanced paternal age may increase the occurrence of adverse pregnancy outcomes such as miscarriage (5), stillbirth (6) or preterm birth (7). The adverse offspring outcomes include higher rates of congenital malformations (8), malignancies (9), early onset schizophrenia (10), autism (11) and other psychiatry or academic morbidities (12).

Birth weight is often considered to be the most direct marker to evaluate fetal growth in utero and intrauterine environment quality. The Developmental Origins of Health and Disease Science clearly suggests that early life growth and development influences health and well-being trajectories into adulthood (13). It has been suggested that advanced paternal age may contribute to the increasing rate of low birth weight infants which may indirectly result in a higher incidence of infant mortality, childhood morbidity (14) and cardiovascular disease in adulthood (15). Because there is an increased risk of preterm birth and very early preterm birth among offspring of men with advanced paternal age, low birth weight may not be an ideal outcome to characterize the effect of advanced paternal age on infant birth weight. Thus, we used neonatal weight for gestational age standard as a more precise evaluation method and conducted this large retrospective study to explore the association between advanced paternal age and birth weight.

## Materials and Methods

### Study Population

**Figure 1:**
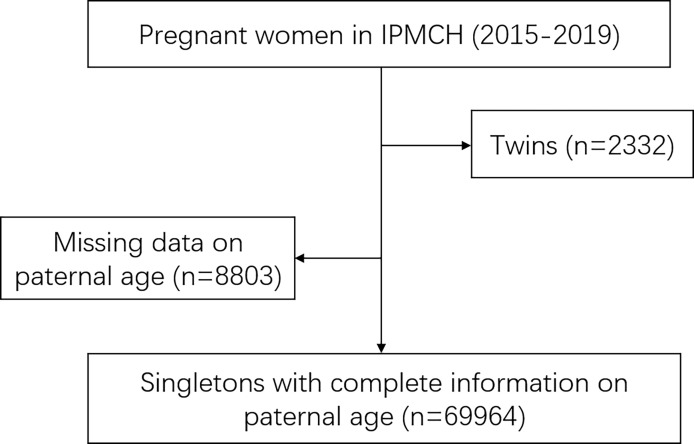
Flow chart: 69,964 cases are enrolled in this study.

This study was performed at the International Peace Maternity and Child Health Hospital located in Shanghai, China. As a hospital delivery population-based cohort study, female participants were those who underwent first-trimester prenatal screenings at the hospital between January 2015 and December 2019. A total of 80,811 women registered at the obstetrics department. After excluding 2,332 cases of twins and 8,803 cases of missing the data of paternal age, 69,964 cases were included in this study (**Figure 1**). All of them had complete data of maternal age, paternal age, gestational week and birth weight. All data are available at the ResMan Manager of Chinese Clinical Trial Registry (www.medresman.org) with the registration number ChiCTR2000038345. Ethics approval for this study was provided by the participating hospital medical ethical committee (no.GKLW2013-9151) and all participants provided written informed consent to have their data included in retrospective analyses.

### Data Collection

All data were collected by hospital nurses, medical residents, and gynecologists with the use of electronic medical record. Parental age, education level and demographic data were collected *via* first clinical presentation. Maternal height and weight were measured to calculate early pregnancy body mass index (BMI) at the first prenatal visit. BMI was used to characterize the women as underweight (BMI <18.5 kg/m^2^), normal (BMI 18.5 to 24.9 kg/m^2^), and overweight or obesity (OWO) (BMI ≥25 kg/m^2^) using the World Health Organization criteria (16). Gestational age was calculated based on the date of last menstrual period and adjusted by ultrasonography in early pregnancy. Hypertension and diabetes mellitus whether preexisting or induced by pregnancy were prospectively recorded in the medical records. Pregnancy-induced hypertension was defined as a newly onset hypertension without proteinuria with a blood pressure higher than 140/90 mmHg after 20 weeks’ gestation (17). Gestational diabetes mellitus was defined according to the diagnostic standard of the American Diabetes Association (18). Preterm birth (PTB) was defined as birth before 37 weeks of gestation. Very early preterm birth (VPTB) was defined as birth before 34 weeks of gestation. Late preterm birth was defined as birth during 34 to 36 weeks of gestation.

The primary outcome of our study was birth weight. Small for gestational age (SGA) was defined as a birth weight less than 10th percentile for gestational age and large for gestation age (LGA) was defined as a birth weight more than 90th percentile for gestational age. The percentiles were based on those published in the INTERGROWTH-21st neonatal weight-for gestational-age standard (19). Information of birth weight, fetal sex and gestational age were recorded on electronic medical files.

### Statistical analysis

We use a multiple logistic regression model to analyze the association of paternal age and multiple birth weight and pregnancy outcomes. Parental age was defined as the father’s age at the time of childbirth. We analyzed parental age as continuous variables and estimated the risk for SGA and LGA associated with a 10-year increase in age. We first used restricted cubic splines with five knots at the fifth, 27.5th, 50th,72.5th and 95th percentiles (20) to assess the potential nonlinear response for continuous age variables and risks of SGA, LGA, LBW and Macrosomia. For consistency with previous studies (7), paternal age variables were grouped into 10-year intervals and categorized into the following four categories: younger than 25 years, 25 to 34 years, 35 to 44 years, and 45 years or older. The reference group was set as 25 to 34 years for categorical age analyses. Continuous variables were described as medians with 95% CI. Categorical variables were represented as frequencies with proportions. Linear regression coefficients and logistic regression odds ratios with 95% confidence intervals were used to estimate pregnancy and infant outcomes in relation to paternal age levels (<25, 25 to 34, 35 to 44, and >44). Potential confounders were considered in multivariable analysis. Covariates, including maternal age (<25, 25 to 34, or >35 years), maternal education years (<9, 10 to 12, 13 to 15, or >16 years), marital status (live with or without fathers), maternal ethnicity (Han or others), maternal BMI (underweight, normal, overweight or obesity), were included to estimate the associations between paternal age and risk for adverse birth weight outcome. Subgroups of women with different age (<24, 25 to 34, or >35 years) and BMI ranges (BMI <18.5 kg/m^2^, 18.5 to 24.9 kg/m^2^, and >25 kg/m^2^) are generated for sensitivity analysis. Paternal age variables which grouped into 5-year intervals are also analyzed for sensitivity analysis and categorized into the following eight categories: younger than 25 years, 25 to 29 years, 30 to 34 years, 35 to 39 years, 40 to 44 years, 45 to 49 years, 50 to 54 years and 55 years or older. Further, we studied the combined effects of maternal BMI and paternal age on birth weight by adding a product interaction term of the maternal BMI × paternal age to the models. The same analysis is also conducted on the combining effects on the occurrence of SGA and LGA. A heat map was constructed to display the differences (red indicates high risk, blue indicates low risk).

For variables with missing data, multiple imputations according to the Markov chain Monte Carlo method were used (21). There were 0.75% cases missing data of maternal BMI. Missing data were imputed using multiple imputation by chained equations with predictive mean matching. Five imputed data sets were generated for analyses. No significant differences in descriptive characteristics were found between the original and imputed data sets. All statistical analyses were performed using R statistical software version 4.0.3 (package rms, visreg), Statistical Package of Social Sciences version 25.0 for Windows (IBM Corp, Armonk, NY) or GraphPad Prism 8.0.1.

## Results

### Population Characteristics

In our analysis, 69,964 cases were included where data files were organized sequentially by year and all available demographic variables, including age, ethnicity, education level, marital status, maternal BMI in early pregnancy, etcetera were extracted. Population characteristics are presented (**Table S1**). The median (95%CI) paternal age was 32.5 (26.0 to 41.0) years with maternal age decreasing slightly to 30.1 (25.0 to 38.0) years. The median (95%CI) birth weight was 3,329.0 (2,640.0 to 4,025.0) g. The study population was primarily nulliparous (71.8%) with a naturally conceived pregnancy (94.6%) and not diagnosed with gestational diabetes (86.3%) or pregnancy-induced hypertension (95.9%). The majority of women (87.4%) had an upper secondary-level education or a bachelor’s degree. Paternal age was categorized into 10-year intervals: <25 (n = 752; 10.7%), 25–34 (n = 48,545; 69.4%), 35–44 (n = 19,263; 27.5%) and 45 or older (n = 1,404; 2.0%). The overall rate of SGA and LGA was 1.8 and 17.6% respectively.

### Paternal Age and the Risk of Adverse Birth Weight and Preterm Birth

**Figure 2:**
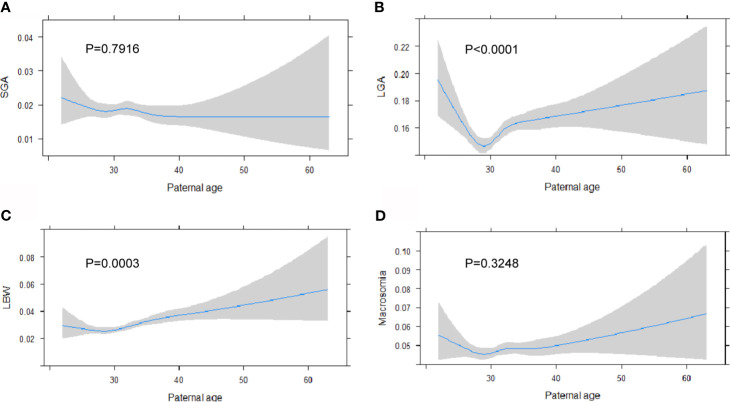
Paternal age is associated with higher risk of adverse birth weight outcomes. Linear regression model is generated for paternal age at birth with the incidence of SGA **(A)**, LGA **(B)**, LBW **(C)** and Macrosomia **(D)**, expressed as predicted prevalence with 95% CIs. Analyses were adjusted for maternal age, BMI, ethnicity, education level and marital status.

Stratified by the four paternal age groups, the occurrence of adverse pregnancy outcomes, including PTB and VPTB as well as gestational complications including gestational diabetes mellitus and pregnancy-induced hypertension, were significantly increased with by aging (**Table 1**). For infant outcomes, a higher risk for a lower Apgar score (<8) and adverse fetal composite (including stillbirth, birth before 28 weeks and SGA less than three percentile) was observed for father older than 35 in comparison to those aged 25–34. As for birth weight, incidence for LGA positively associated with paternal age while SGA was negatively associated. Using linear regression modeling to study the association between paternal age and multiple birth weight outcomes, there is an observational U-shaped nonlinear association between LGA (P <.001) and LBW (P <.001) with paternal age (**Figures 2A–D**). Further, gestational age was lower among infants born to fathers aged 35–44 by an average of 0.11 weeks (95% CI, −0.14 to −0.08 weeks) and fathers with advanced age were 13% more likely to have a PTB compared with younger fathers (OR, 1.13; 95%CI, 1.03 to 1.24, **Table 2**). Similarly, fathers who were 45 years of age and older had infant born 0.15 weeks younger (95%CI, −0.23 to −0.06) and were 36% more likely to have a PTB compared with younger fathers (OR, 1.36; 95%CI, 1.09 to 1.70). Partners of fathers with advanced age also had increased rates of gestational diabetes and pregnancy induced hypertension although this is not reach statistical significance. Lastly, infants born to fathers aged 34–44 were 23% more likely to be LBW than those born to younger fathers (OR, 1.23; 95% CI, 1.08 to 1.39) and were 7% more likely to be LGA (OR, 1.07; 95%CI, 1.01 to 1.13). There was no significant difference in SGA between the four paternal age groups (**Table 2**).

**Table 1:** Maternal characteristics, pregnancy and infant outcomes by paternal age group.

	Paternal age (years)			
**Maternal characteristics**	<25 (n = 752)	25–34 (n = 48,545)	35–44 (n = 19,263)	>44 (n = 1,404)
Maternal age (mean, 95%CI)	24.0 (22.00–29.00)	29.0 (25.0–34.0)	35.0 (29.0–40.0)	37.0 (29.0–43.0)
Maternal BMI (mean, 95%CI)	19.9 (16.70–25.94)	20.6 (17.4–26.0)	21.3 (17.9–26.9)	21.3 (18.0–26.7)
**Pregnancy outcomes**				
Gestational weeks (mean, 95%CI)	39.30 (36.8–40.0)	39.20 (37.0–41.0)	38.60 (36.4–40.5)	38.60 (36.2–40.4)
Preterm birth (n, %)	37 (4.9)	2,328 (4.8)	1,226 (6.4)	110 (7.8)
Very early preterm birth (n, %)	7 (0.9)	382 (0.8)	274 (1.4)	26 (1.9)
Gestational diabetes (n, %)	58 (7.7)	5,693 (11.7)	3,523 (18.3)	321 (22.9)
Gestational hypertension (n, %)	27 (3.6)	1,806 (3.7)	926 (4.8)	87 (6.2)
**Infant outcomes**				
Birth weight (mean, 95%CI)	3,340 (2,641.5–4,057.0)	3,330 (2,650.0–4,020.0)	3,345 (2,610–4,035)	3,311 (2,496.2–4,057.5)
Low birth weight (n, %)	25 (3.3)	1,315 (2.7)	676 (3.5)	70 (4.9)
Macrosomia (>4,000 g)	46 (6.1)	2,588 (5.3)	1,115 (5.8)	86 (6.1)
Small for gestational age (10%), (n, %)	20 (2.7)	918 (1.9)	304 (1.6)	18 (1.3)
Large for gestational age (90%), (n, %)	127 (16.9)	7,939 (16.4)	3,947 (20.5)	296 (21.0)
Low 5-minute Apgar score (<8), (n, %)	23 (3.1)	1,374 (2.8)	682 (3.5)	55 (3.9)
Adverse fetal composite, (n, %)	1 (0.1)	191 (0.4)	82 (0.4)	6 (0.4)

Values are numbers (percentages) unless stated otherwise. Adverse fetal composite: stillbirth, delivery earlier than 28 weeks, birth weight less than the third percentile for gestational age and sex.

**Table 2:** Unadjusted and adjusted risk of major outcomes stratified by paternal age in 69,964 pregnancies.

	Paternal age (years)			
**Unadjusted model, OR (95% CI)**	<25 (n = 752)	25–34 (n = 48,545)	35–44 (n = 19,263)	>44 (n = 1,404)
Gestational weeks (weeks)	0.13 (0.03 to 0.23)	reference	−0.31 (−0.34 to −0.29)	−0.44 (−0.52 to −0.37)
Preterm birth (<37** w**)	1.03 (0.74 to 1.43)	reference	1.35 (1.26 to 1.45)	1.69 (1.38 to 2.06)
Very early preterm birth (<34** w**)	1.19 (0.56 to 2.51)	reference	1.82 (1.56 to 2.13)	2.38 (1.59 to 3.55)
Birth weight (mean, 95%CI)	3.17 (-28.69 to 35.04)	reference	3.01 (−4.37 to 10.40)	−17.39 (−40.88 to 6.08)
Low birth weight (<2,500 g)	1.24 (0.83 to 1.85)	reference	1.31 (1.19 to 1.44)	1.89 (1.47 to 2.41)
Small for gestational age (10%)	1.42 (0.91 to 2.22)	reference	0.83 (0.73 to 0.95)	0.67 (0.42 to 1.08)
Large for gestational age (90%)	1.04 (0.86 to 1.26)	reference	1.32 (1.26 to 1.38)	1.37 (1.20 to 1.56)
Macrosomia (>4,000 g)	1.16 (0.86 to 1.56)	reference	1.09 (1.02 to 1.17)	1.16 (0.93 to 1.45)
Low 5-minute Apgar score (<8)	1.08 (0.71 to 1.65)	reference	1.26 (1.15 to 1.38)	1.40 (1.06 to 1.84)
Adverse fetal composite, (n, %)	0.34 (0.05 to 2.41)	reference	1.08 (0.84 to 1.40)	1.09 (0.48 to 2.45)
**Adjusted model, OR (95% CI)**				
Gestational weeks (weeks)	−0.40 (−0.14 to 0.07)	reference	−0.11 (−0.14 to −0.08)	−0.15 (−0.23 to −0.06)
Preterm birth (<37** w**)	1.21 (0.86 to 1.70)	reference	1.13 (1.03 to 1.24)	1.36 (1.09 to 1.70)
Very early preterm birth (<34** w**)	1.63 (0.76 to 3.48)	reference	1.37 (1.12 to 1.69)	1.55 (0.98 to 2.47)
Birth weight (mean, 95%CI)	13.11 (−19.51 to 45.72)	reference	−5.83 (−15.27 to 3.60)	−14.34 (−39.70 to 11.02)
Low birth weight (<2,500 g)	1.32 (0.87 to 2.01)	reference	1.23 (1.08 to 1.39)	1.65 (1.25 to 2.19)
Small for gestational age (10%)	1.29 (0.81 to 2.07)	reference	0.92 (0.78 to 1.08)	0.69 (0.41 to 1.16)
Large for gestational age (90%)	1.28 (1.05 to 1.57)	reference	1.07 (1.01 to 1.13)	1.10 (0.94 to 1.26)
Macrosomia (>4,000 g)	1.26 (0.92 to 1.72)	reference	1.01 (0.91 to 1.11)	1.14 (0.90 to 1.46)
Low 5-minute Apgar score (<8)	1.24 (0.68 to 2.28)	reference	1.14 (0.96 to 1.35)	1.17 (0.75 to 1.80)
Adverse fetal composite, (n, %)	0.345 (0.05 to 2.49)	reference	1.241 (0.88 to 1.74)	1.113 (0.43 to 2.85)

Analyses were adjusted for maternal age, BMI, ethnicity, education level and marital status. Values are linear regression coefficients or logistic regression odds ratios with 95% confidence intervals. Adverse fetal composite: stillbirth, delivery earlier than 28 weeks, birth weight less than the third percentile for gestational age and sex.

### The Association Between Paternal Age and Birth Weight Differ in Preterm and Term Birth

**Figure 3:**
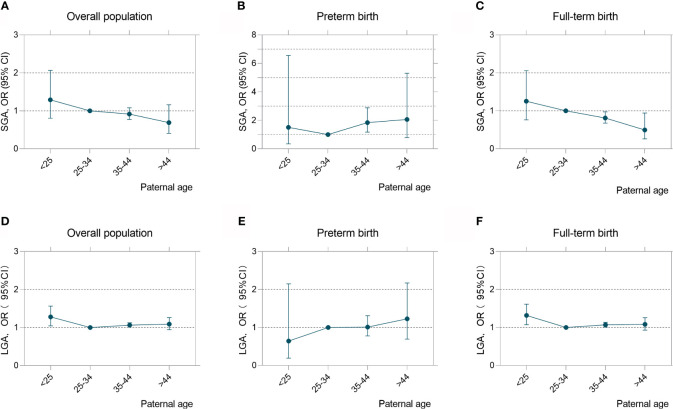
Paternal age exerted an opposite effect on birthweight among preterm and term infants. Adjusted odds ratios (ORs) and 95% confidence intervals for risks of SGA **(A–C)** and LGA **(D–F)** according to paternal age in overall population, preterm birth and full-term birth infants. Analyses were adjusted for maternal age, BMI, ethnicity, education level and marital status.

Given preterm birth and LGA rates increased with paternal age but not the incidence of SGA, we examined if there may be differences in risk for SGA and LGA among infants born term and preterm. As shown in **Figure 3**, paternal age exhibited dichotomous effect on birth weight with increased risk of SGA (35–44years: OR = 1.85, 95%CI (1.18 to 2.89) among infants born preterm birth and a decreased risk for infants born at term (35–44years: OR, 0.81, 95%CI (0.68 to 0.97); >44years: OR, 0.50, 95%CI (0.26 to 0.94). U-shaped associations were found where LGA risk was higher among young fathers (<25 years) (OR, 1.32; 95% CI, 1.07 to 1.62) and those who are older (35–44 years) (OR, 1.07; 95% CI, 1.01 to 1.14) in comparison to infant born among fathers who were 25 to 34 years old at delivery (**Table S2**). This result is consistency with the outcomes when stratified paternal age in a 5-year interval (**Table S3**). When analyzing paternal age as a continuous variable and examining the linear association between SGA and LGA rates by the three gestational age subgroups (**Table 3**), there was no increased risk for SGA or LGA among VPTB infants. However, per-year increases in paternal age were associated with an increased risk for SGA among late preterm birth infants (adjusted OR, 1.07; 95% CI, 1.01 to 1.13) and a decreased risk among full-term infants (adjusted OR, 0.99; 95%CI, 0.97 to 1.00). A per-unit increase in paternal age was also associated with an increased risk for LGA among full-term infants but not preterm infants (adjusted OR, 1.01; 95% CI, 1.01 to 1.02).

**Table 3:** Per-Unit Increase of paternal age and Risks of SGA and LGA by preterm and full-term birth group.

	Very early preterm birth: <34 w (n = 688)	Late preterm birth: 34–36w (n = 3,017)	Full-term birth: ≥37 w (n = 66,259)
**SGA**			
n (%)	78 (11.30)	71 (2.40)	1,111 (1.70)
OR (95%CI)	1.01 (0.96 to 1.05)	1.03 (0.98 to 1.07)	0.97 (0.96 to 0.98)
Adjusted OR (95%CI)	0.96 (0.90 to 1.03)	1.07 (1.01 to 1.13)	0.99 (0.97 to 1.00)
**LGA**			
n (%)	142 (20.60)	412 (13.70)	11,755 (17.70)
OR (95%CI)	0.99 (0.96 to 1.03)	1.01 (0.99 to 1.03)	1.03 (1.03 to 1.04)
Adjusted OR (95%CI)	0.99 (0.94 to 1.05)	1.00 (0.96 to 1.03)	1.01 (1.01 to 1.02)

Adjusted for maternal age, BMI, ethnicity, education level and marital status; per-unit increase are considered as one year increase in paternal age.

### Combined Effects of Paternal Age and Maternal BMI on the Outcome of Birth Weight

**Figure 4:**
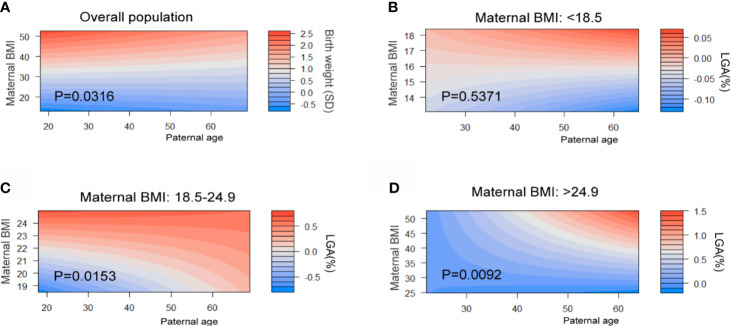
Combined effects of maternal BMI in early pregnancy and paternal age on birth weight and LGA. Maternal BMI were categorized into three stages: underweight (BMI <18.5 kg/m^2^), normal (BMI 18.5 to 24.9 kg/m^2^), and overweight or obesity (OWO) (BMI ≥25 kg/m^2^). Heat map (filled contour plot) for the correlation of gestational age–adjusted birth weight **(A)** and paternal age in overall population (red indicates increased gestational age–adjusted birth weight; blue indicates decreased gestational age–adjusted birth weight). Heat map for the prevalence of LGA in three maternal BMI levels **(B–D)** (red indicates high risk, blue indicates low risk) according to their interaction with maternal BMI and paternal age are generated. Analyses were adjusted for maternal age, BMI, ethnicity, education level and marital status.

Maternal BMI in early pregnancy have a crucial impact on the birth weight outcome. From the results we describe above, we hypothesized that paternal age has greater effects on birth weight as well as the incidence of SGA and LGA. Therefore, we studied the combined effects of two factors. Standardized birth weights adjusted by gestational age are generated for the next analysis. A heatmap (filled contour plot) for the combined association of maternal BMI (y-axis) and paternal age (x-axis) with birth weight (SD) (z-axis; red indicates higher birth weight, blue indicates lower birth weight) was plotted (**Figure 4**). We generate the heatmap of overall population and three subgroups categorized by maternal BMI. First, in overall populations, the interaction between paternal age and maternal BMI was significant for the birth weight for gestational age (P = 0.032). When stratified by maternal BMI, the interaction contribution of SGA did not reach significant difference (**Figure S4**). Conversely, the effect estimates for paternal age differ in LGA by stratifications. Considerable combing effects of two factors on higher risk of LGA in two subgroups are also identified (BMI: 18.5–24.9, P for interaction = 0.015; BMI >24.9, P for interaction = 0.009, **Figures 4C, D**).

### Sensitivity Analysis

To further investigate the association between paternal age and birth weight, stratification by multiple maternal factors were analyzed. First, after stratification by maternal age, paternal age remained significantly associated with SGA and LGA (**Tables S4**, **S5**). When maternal age was between 25 and 34 years, the adjusted odds ratio of older than 45 years was 1.48 with 95% CI, 1.14 to 1.91 compared to the reference group in LGA (**Figure S3**). When maternal BMI was in the normal range (18.5–24.9 kg/m^2^), fathers aged 35–44 years had a 9% (OR, 1.09; 95%CI, 1.02 to 1.16) and those aged >45 years had a 15% (OR, 1.15; 95%CI, 0.98 to 1.35) increased likelihood of having a LGA infant in comparison to younger fathers (**Figure S4**). Similarly, the risk for SGA decreased with increasing paternal age with exception when mother was in obesity or overweight status (**Figure S4**). When categorized by IVF and parity, the same trend was observed among the non-IVF and nulliparous populations while in the group of women with IVF treatment and multiple parity a significant difference was not found. Similar sensitivity analysis was also conducted in paternal age grouped in a 5-year interval (**Tables S6**, **S7**).

## Discussion

### Main Findings

In this large retrospective population-based cohort study examining over 69,000 hospital deliveries we found paternal age has a significant effect on birth weight with advanced paternal age increasing the risk of SGA among preterm infants and decreasing the risk among infants born at term. U-shaped associations were found in that LGA risk was higher among father <25 years and those who are older between 35 and 44 years of age especially among term infants when compared to infants whose fathers were aged 25 to 34 years of age.

We studied the association between paternal age and birth weight in multiple linear regression model and observed a strong U-shape association between paternal age and LGA in the overall cohort population. Due to the coexisting risk of LGA and LBW, we considered that high preterm birth risks may be a possible confounding factor when examining the association between paternal age and birth weight. When we categorized the population gestational weeks to observe the potential different impact of paternal age on birth weight, paternal age demonstrated dichotomous effect on birth weight with an increased risk of SGA among preterm and late preterm infants and a decreased risk among full-term infants. LGA risk was significantly higher among both younger (<25 years) and older (35–44 years) fathers when compared to infants whose fathers were aged 25 to 34 years in the overall population and the full-term subgroup. Further, maternal BMI and paternal effect has a combined effect on the prevalence of LGA. These results suggest the effect of advanced paternal age on birth weight is complicated and should be evaluated according to clinical circumstances.

### Interpretations

A significant number of studies have examined the association between maternal characteristics and child outcomes while few have examined paternal factors. Advanced paternal age has been reported to increase the risk for adverse pregnancy and neonatal outcomes such as low birth weight and preterm birth (7). It also may exert a long-term negative impact on the child into adulthood including metabolic risks (22) and psychological disease (11, 12). Preliminary evidence also suggests aging fathers may enhance the mortality (9) and shorten the life expectancy of their child (23). One pathway may be related to research indicating paternal age may be associated with adverse lipid profiles (24) and possibly an elevated risk for obesity in young adulthood (22). Neonatal birth weight as a crucial marker to evaluate fetal growth and reflects an intrauterine environment which can have a significant effect on adulthood heath. Large gestation age (LGA) is as an early-life trait of adulthood obesity and diabetes (25). However, few studies have been focused on studying the association between paternal age and birth weight adjusted by gestational age. Our study addresses an important clinical gap between paternal factors and child outcomes by clearly outlining a positive association between advanced paternal age and LGA among term infants. Besides, small gestation age (SGA) is a crucial indicator of infant congenital abnormality (8) and mortality (26, 27). Researchers also found that preterm children born as SGA also had a higher risk of obesity when comparing to those born as appropriate for gestational age in childhood (28). This interesting phenomenon can be explained by rapid compensational weight gaining in infancy (29, 30). Those born SGA and develop high BMI by catch-up growth in childhood are at increased risk of abnormal glucose metabolism in adulthood (31).

Maternal age and BMI are two determinant factors for the development of LGA (32). As such, we divided 69,964 cases into subgroups stratified by maternal age and maternal BMI individually in order to examine whether the association between paternal age and birth weight is stable. We found that when maternal age is in a normal range (25–34), the infants of aging fathers are at a higher risk for LGA. A significantly elevated risks for LGA contributed by advanced paternal age was confirmed among a subgroup of normal-BMI women. However, we did not observe significance difference in GDM risk among four paternal age groups though GDM is considered as a crucial complication contribute to a higher risk of LGA (33).These results strengthened the independent effect of advanced paternal age on LGA.

When considering the underlying mechanism of this phenomenon, previous research involving mouse models suggests that advanced paternal age could exert adverse metabolic effects on offspring by impairing glucose tolerance (34). One possible explanation is that aging exerts epigenetic changes in male sperm including methylation status and histone modifications which may be an explanation for its effect on an offspring’s increased risk for metabolism health conditions (23).

### Strength and Limitations

The major strength of the current study is the rigorous examination of the association between advanced paternal age and birth weight for gestational age for the first time. Complete medical information allowed us to study maternal confounding factors such as age, educational level, marital status and early pregnancy BMI. However, several limitations should be noted. First, this study was a hospital-based cohort, and while the homogeneous ethnicity of the cohort increased the internal validity it decreased the external validity. Second, due to the limited paternal demographic data, our conclusion requires future studies to exclude paternal confounding factors other than age. Third, among the population of non-nullipara women, comparing the pregnancy and fetal outcome within family can minimized the unobserved parental characteristics (35). A sibling fixed model can assist in confirming our results.

In conclusion, our results suggest that paternal age is associated with birth weight. Paternal age had a significant effect on infant birth weight with an increased risk for SGA among preterm infants and LGA among term infants. These findings likely reflect a pathophysiology pathway and have clinical implications for prenatal counseling and pediatric care. Future studies including prospective cohorts or randomized controlled trials are required to confirm the developmental effect of advanced paternal age on child in order to guide preventative interventions to minimize adverse fetal outcomes. Elucidating the mechanisms of how male aging can influence infant growth trajectories in clinically important given more families are selecting to have children at an advanced age.

## Data Availability Statement

All data are available at the ResMan Manager of Chinese Clinical Trial Registry (www.medresman.org) with the registration number ChiCTR2000038345.

## Ethics Statement

The studies involving human participants were reviewed and approved by the participating hospital medical ethical committee (no.GKLW2013-9151) and all participants provided written informed consent to have their data included in retrospective analyses. The patients/participants provided their written informed consent to participate in this study.

## Author Contributions

HH and YWu had full access to all the data in the study and takes responsibility for the integrity of the data and the accuracy of the data analysis. Concept and design: YTM, CZ, and YWu. Acquisition, analysis, or interpretation of data: YTM, CZ, YWa, YCM and JS. Drafting of the manuscript: YTM. Revision of the manuscript: YWu and C-LD. Supervision: HH. All authors contributed to the article and approved the submitted version.

## Funding

This research is supported by National Natural Science Foundation of China (81661128010,82001571, 81671412), the International Science and Technology Collaborative Fund of Shanghai (18410711800), Program of Shanghai Academic Research Leader (20XD1424100), Outstanding Youth Medical Talents of Shanghai Rising Stars of Medical Talent Youth Development Program, Science and Technology Innovation Fund of Shanghai Jiao Tong University (YG2019GD04, YG2020YQ29), Clinical Research Plan of Shanghai Shenkang Hospital Development Center (SHDC12018X17, SHDC12019107), CAMS Innovation Fund for Medical Sciences (2019-12M-5-064), and Clinical Research Project of Shanghai Municipal Health Commission (201840210, 20184Y0349).

## Conflict of Interest

The authors declare that the research was conducted in the absence of any commercial or financial relationships that could be construed as a potential conflict of interest.
