# Supplementary material for: Association Between Paternal Age and Birth Weight in Preterm and Full-Term Birth: A Retrospective Study
Source: Front Endocrinol (Lausanne). 2021 Jul 22;12:706369. doi: 10.3389/fendo.2021.706369 (PMC8341720; doi:10.3389/fendo.2021.706369)
Supplement: Supplementary file 1 [file DataSheet_1.docx]

**Title:** Association between Paternal age and Birthweight in preterm and full-term birth: A retrospective study

Yiting Mao^1,4, *^, MD; Chen Zhang^1, 2, *^, MPH; Yinyu Wang^1,4^, PhD; Yicong Meng^1,4^, PhD; Lei Chen^1^, MSC; Cindy-Lee Dennis, PhD^5^; Jianzhong Sheng^4.6^, PhD; Yanting Wu^2, #^, PhD; Hefeng Huang^1,3 4, #^, MD

**Supplementary:**

Figure S1 Adjusted odds Ratios (ORs) and 95% Confidence Intervals for risks of SGA and LGA according to paternal age in overall population, preterm birth and full-term birth (Grouped in 5 years interval)

Figure S2 Sensitivity analysis: Adjusted odds Ratios (ORs) and 95% Confidence Intervals for risks of SGA and LGA with paternal age stratified by maternal age

Figure S3 Sensitivity analysis: Adjusted odds Ratios (ORs) and 95% Confidence Intervals for risks of SGA and LGA with paternal age stratified by maternal BMI

Figure S4: Combined effects of maternal BMI in early pregnancy and paternal age on SGA. Maternal BMI were categorized into three stages: underweight (BMI <18.5 kg/m^2^), normal (BMI 18.5 to 24.9 kg/m^2^), and overweight or obesity (OWO) (BMI≥25 kg/m^2^). Heat map (filled contour plot) for the correlation of prevalence of SGA (red indicates high risk, blue indicates low risk) according to their interaction with three maternal BMI categories and paternal age are generated. Analyses were adjusted for maternal age, BMI, ethnicity, education level and marital status.

Table S1 Population characteristics of 69964 cases enrolled

Table S2 Paternal age and risks of SGA and LGA stratified by preterm birth and full-term birth at 10 years interval

Table S3 Paternal age and risks of SGA and LGA stratified by preterm birth and full-term birth at 5 years interval

Table S4 Sensitivity analysis for the association between paternal age and SGA at 10 years interval

Table S5 Sensitivity analysis for the association between paternal age and LGA at 10 years interval

Table S6 Sensitivity analysis for association between paternal age and SGA at 5 years interval

Table S7 Sensitivity analysis for association between paternal age and LGA at 5 years interval

Figure S1 Adjusted odds Ratios (ORs) and 95% Confidence Intervals for risks of SGA and LGA according to paternal age in overall population, preterm birth and full-term birth (Grouped in 5 years interval).


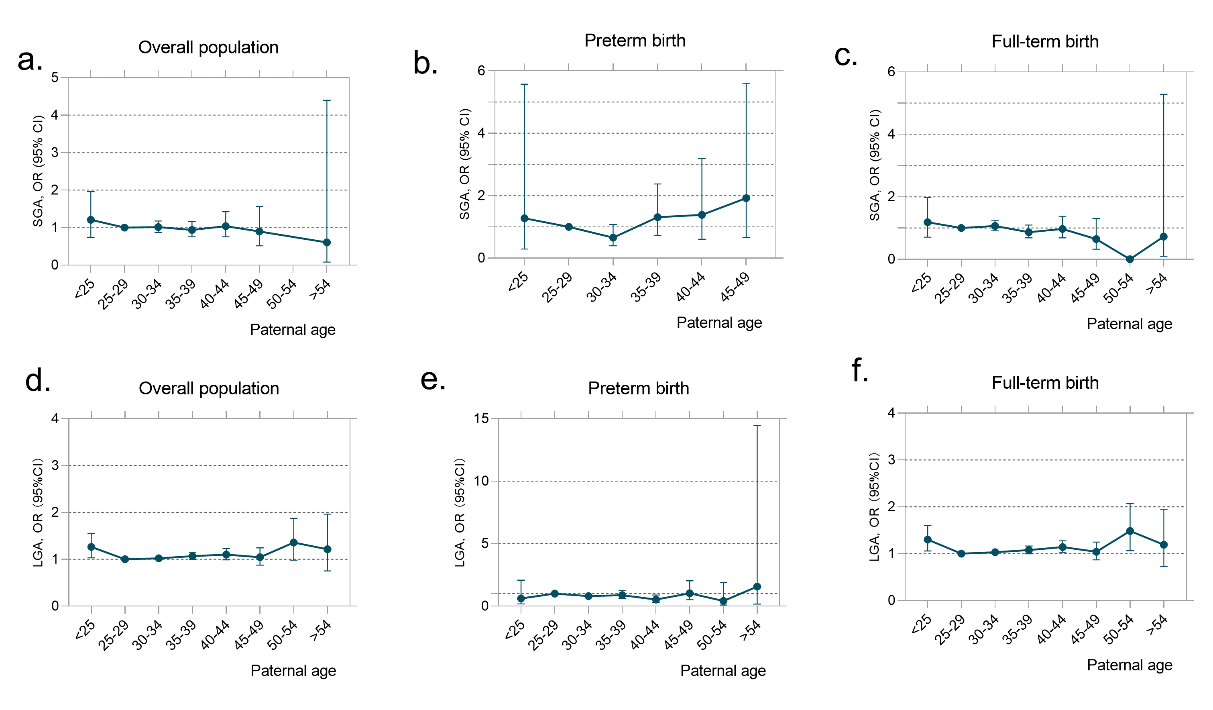


Figure S2 Sensitivity analysis: Adjusted odds Ratios (ORs) and 95% Confidence Intervals for risks of SGA and LGA with paternal age stratified by maternal age


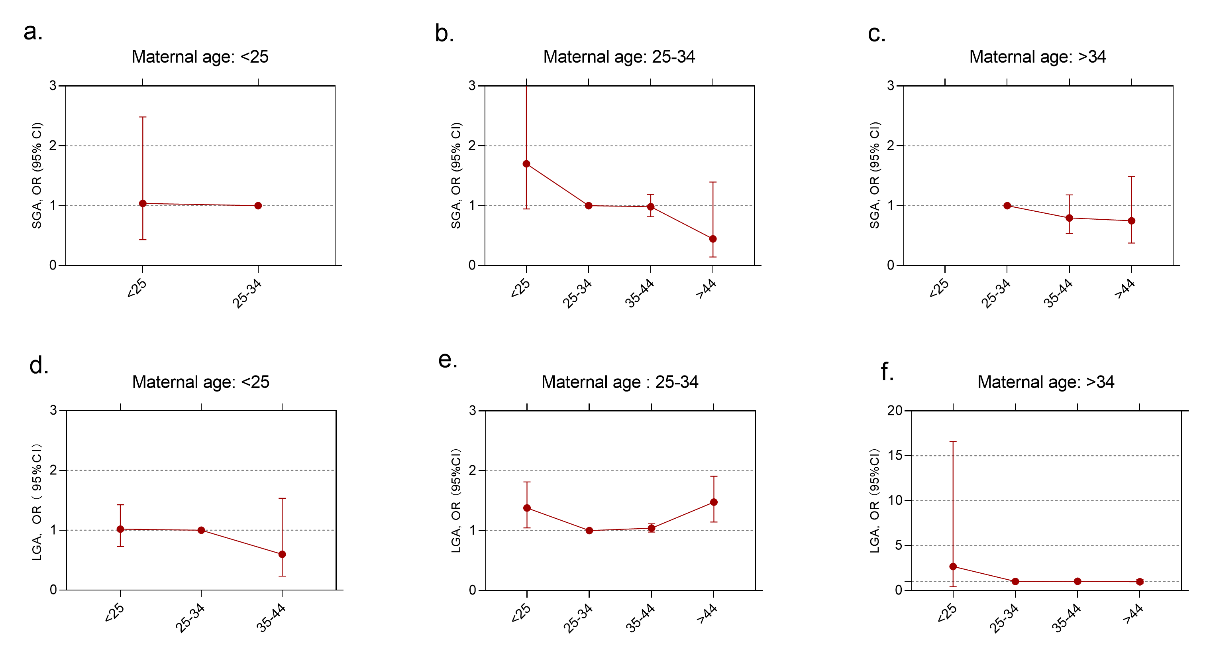


Figure S3 Sensitivity analysis: Adjusted odds Ratios (ORs) and 95% Confidence Intervals for risks of SGA and LGA with paternal age stratified by maternal BMI


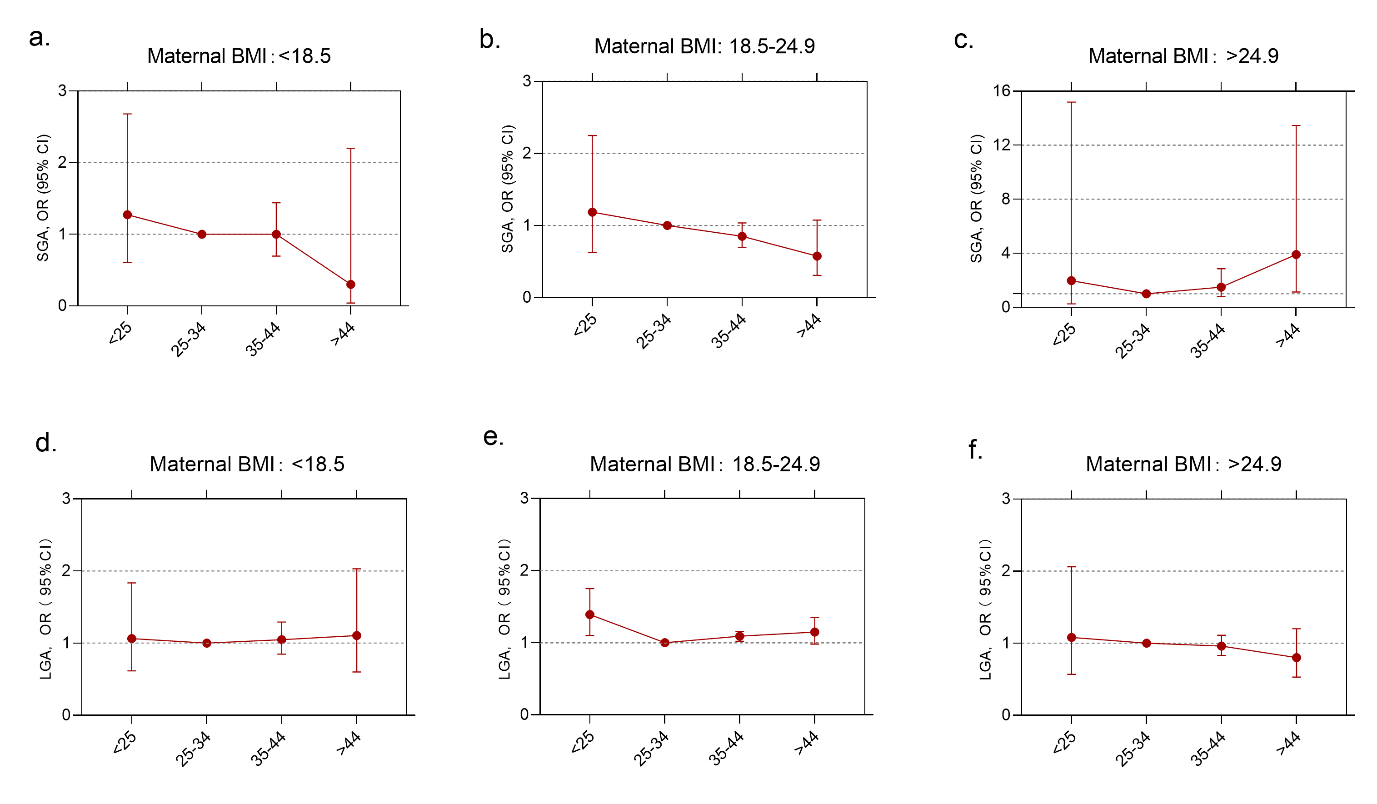


Table S1 Population characteristics of 69964 cases enrolled

| **Maternal Characteristics** | **Overall population** |
| --- | --- |
| Age, mean (95% CI), y | 30.1（25-38） |
| <20 | 1 (0.1) |
| 20~29 | 28264 (40.4) |
| 30~39 | 40023 (57.2) |
| ≥40 | 1676 (2.4) |
| Ethnicity |  |
| Han Chinese | 68814 (98.4) |
| Others | 1150 (1.6) |
| BMI, mean (95% CI), kg/m^2^ | 21.19（17.5-26.3） |
| underweight (<18.5) | 9062 (13.0) |
| normal (18.5-24.9) | 54421 (77.8) |
| overweight or obesity (>24.9) | 6481 (9.3) |
| Nullipara, n (%) | 50226 (71.8) |
| Marital status, n (%) |  |
| Married | 69371 (99.2) |
| Others | 593 (0.8) |
| Educational attainment, years |  |
| ≤9 | 1523 (2.2) |
| 10~12 | 3790 (5.4) |
| 13-16 | 47984 (68.6) |
| ≥17 | 13162 (18.8) |
| unknown | 3505 (5.0) |
| IVF fertility treatment (n, %) | 3797 (5.4) |
| Hypertention |  |
| Pregnancy induced | 2846 (4.1) |
| Preexisting | 359 (0.5) |
| Diabetes |  |
| Pregnancy induced | 9595 (13.7) |
| Preexisting | 403 (0.57) |
| **Paternal Characteristics** |  |
| Age, median (95% CI), y | 32.6（26-41） |
| <25 | 752 (1.1) |
| 25~34 | 48545 (69.4) |
| 35~44 | 19263 (27.5) |
| ≥45 | 1404 (2.1) |
| **Fetal Characteristics** |  |
| Birthweight, median (95% CI), g | 3329.0（2640-4025） |
| SGA, n (%) | 1260 (1.8) |
| LGA, n (%) | 12309 (17.6) |
| Male fetal sex, n (%) | 36043 (51.5) |

Table S2 Paternal age and risks of SGA and LGA stratified by preterm birth and full-term birth at 10 years interval

| **SGA: adjusted model, AOR (95% CI)** | <25 | 25-34 | 35-44 | >44 |
| --- | --- | --- | --- | --- |
| Preterm birth | 1.51 (0.35 to 6.56) | reference | 1.85 (1.18 to 2.89) | 2.06 (0.80 to 5.31) |
| Very early preterm birth: <34w | 1.73 (0.20 to 15.18) | reference | 0.92 (0.48 to 1.77) | 1.66 (0.50 to 5.54) |
| Late preterm birth: 34-36w | 1.02 (0.13 to 7.93) | reference | 3.05 (1.63 to 5.70) | 1.24 (0.16 to 9.77) |
| Full term birth: ≥37w | 1.25 (0.76 to 2.06) | reference | 0.81 (0.68 to 0.97) | 0.50 (0.26 to 0.94) |
| **LGA: adjusted model, AOR (95% CI)** |  |  |  |  |
| Preterm birth | 0.64 (0.19 to 2.15) | reference | 1.01 (0.78 ot 1.31) | 1.23 (0.69 to 2.18) |
| Very early preterm birth: <34w | 0.74 (0.09 to 6.37) | reference | 0.84 (0.50 to 1.43) | 1.53 (0.53 to 4.42) |
| Late preterm birth: 34-36w | 0.57 (0.13 to 2.46) | reference | 1.01 (0.75 to 1.36) | 1.22 (0.63 to 2.35) |
| Full term birth: ≥37w | 1.32 (1.07 to 1.62) | reference | 1.07 (1.01 to 1.14) | 1.08 (0.93 to 1.26) |

Table S3 Paternal age and risks of SGA and LGA stratified by preterm birth and full-term birth at 5 years interval

| **SGA: adjusted model, OR (95% CI)** | <25 | 25-29 | 30-34 | 35-39 | 40-44 | 45-49 | 50-54 | >54 |
| --- | --- | --- | --- | --- | --- | --- | --- | --- |
| Preterm birth | 1.28 (0.30 to 5.57) | reference | 0.66 (0.40 to 1.08) | 1.31 (0.73 to 2.38) | 1.39 (0.60 to 3.19) | 1.92 (0.66 to 5.60) | - | - |
| Very early preterm birth: <34w | 1.17 (0.13 to 10.46) | reference | 0.39 (0.18 to 0.81) | 0.41 (0.17 to 1.02) | 0.247 (0.06 to 0.94) | 0.766 (0.17 to 3.46) | - | - |
| Late preterm birth: 34-36w | 0.96 (0.12 to 7.58) | reference | 0.93 (0.46 to 1.88) | 2.72 (1.21 to 6.12) | 4.36 (1.44 to 13.25) | 1.66 (0.19 to 14.19) | - | - |
| Full term birth: ≥37w | 1.18 (0.71 to 1.98) | reference | 1.07 (0.91 to 1.25) | 0.87 (0.69 to 1.10) | 1.00 (0.71 to 1.41) | 0.65 (0.33 to 1.31) | - | 0.73 (0.10 to 5.31) |
| **LGA: adjusted model, OR (95% CI)** |  |  |  |  |  |  |  |  |
| Preterm birth | 0.62 (0.18 to 2.08) | reference | 0.81 (0.62 to 1.06) | 0.88 (0.62 to 1.24) | 0.53 (0.31 to 0.90) | 1.03 (0.52 to 2.03) | 0.41 (0.09 to 1.91) | 1.57 (0.17 to 14.47) |
| Very early preterm birth: <34w | 0.74 (0.08 to 6.46) | reference | 0.89 (0.50 to 1.61) | 0.83 (0.40 to 1.73) | 0.38 (0.12 to 1.22) | 1.34 (0.35 to 5.06) | 0.99 (0.09 to 11.26) | - |
| Late preterm birth: 34-36w | 0.54 (0.13 to 2.35) | reference | 0.80 (0.60 to 1.08) | 0.88 (0.60 to 1.31) | 0.59 (0.32 to 1.08) | 1.1 (0.51 to 2.38) | 0.26 (0.03 to 2.06) | 2.28 (0.23 to 22.72) |
| Full term birth: ≥37w | 1.30 (1.06 to 1.60) | reference | 1.03 (0.97 to 1.09) | 1.08 (1.00 to 1.16) | 1.14 (1.02 to 1.28) | 1.03 (0.86 to 1.23) | 1.49 (1.07 to 2.07) | 1.19 (0.73 to 1.95) |

Table S4 Sensitivity analysis for the association between paternal age and SGA at 10 years interval

| **Stratification** | <25 | 25-34 | 35-44 | >44 |
| --- | --- | --- | --- | --- |
| **Maternal Age: (years)** |  |  |  |  |
| Maternal age: <25 | 1.04 (0.43 to 2.48) | reference | - | - |
| Maternal age: 25-34 | 1.70 (0.95 to 3.06) | reference | 0.98 (0.82 to 1.19) | 0.45 (0.14 to 1.40) |
| Maternal age: >34 | - | reference | 0.80 (0.54 to 1.18) | 0.75 (0.38 to 1.49) |
| **Maternal BMI: kg/m^2^** |  |  |  |  |
| underweight (<18.5) | 1.28 (0.61 to 2.68) | reference | 1.00 (0.70 to 1.44) | 0.30 (0.04 to 2.20) |
| normal (18.5-24.9) | 1.19 (0.63 to 2.25) | reference | 0.85 (0.70 to 1.04) | 0.58 (0.31 to 1.08) |
| overweight or obesity (>24.9) | 1.98 (0.26 to 15.19) | reference | 1.50 (0.79 to 2.86) | 3.91 (1.14 to 13.46) |
| **IVF** |  |  |  |  |
| Non-IVF | 1.29 (0.81 to 2.06) | reference | 0.95 (0.80 to 1.13 ) | 0.80 (0.47 to 1.36) |
| IVF | - | reference | 0.51 (0.28 to 0.94) | 0.15 (0.02 to 1.17) |
| **Parity** |  |  |  |  |
| Nullipara | 1.40 (0.88 to 2.24) | reference | 0.95 (0.79 to 1.15) | 0.59 (0.32 to 1.10) |
| Non-nullipara | - | reference | 1.44 (0.97 to 2.15) | 1.14 (0.42 to 3.05) |

Table S5 Sensitivity analysis for the association between paternal age and LGA at 10 years interval

| **Stratification** | <25 | 25-34 | 35-44 | >44 |
| --- | --- | --- | --- | --- |
| **Maternal Age** |  |  |  |  |
| Maternal age: <25 | 1.02 (0.73 to 1.43) | reference | 0.60 (0.23 to 1.53) | - |
| Maternal age: 25-34 | 1.38 (1.05 to 1.81) | reference | 1.04 (0.98 to 1.11) | 1.48 (1.14 to 1.91) |
| Maternal age: >34 | 2.67 (0.43 to 16.59) | reference | 1.03 (0.91 to 1.17) | 0.98 (0.80 to 1.20) |
| **Maternal BMI: kg/m^2^** |  |  |  |  |
| underweight (<18.5) | 1.06 (0.62 to 1.84) | reference | 1.05 (0.85 to 1.29) | 1.11 (0.60 to 2.03) |
| normal (18.5-24.9) | 1.39 (1.10 to 1.75) | reference | 1.09 (1.02 to 1.16) | 1.15 (0.98 to 1.35) |
| overweight or obesity (>24.9) | 1.08 (0.57 to 2.06) | reference | 0.96 (0.83 to 1.11) | 0.80 (0.53 to 1.20) |
| **IVF** |  |  |  |  |
| Non-IVF | 1.32 (1.08 to 1.61) | reference | 1.08 (1.02 to 1.14) | 1.06 (0.90 to 1.23) |
| IVF | - | reference | 0.91 (0.75 to 1.11) | 1.32 (0.88 to 1.97) |
| **Parity** |  |  |  |  |
| Nullipara | 1.30 (1.05 to 1.60) | reference | 1.01 (0.94 to 1.09) | 1.20 (0.98 to 1.48) |
| Non-nullipara | 0.65 (0.25 to 1.68) | reference | 0.99 (0.91 to 1.08) | 1.02 (0.83 to 1.25) |

Table S6 Sensitivity analysis for association between paternal age and SGA at 5 years interval

| **SGA: adjusted model, OR (95% CI)** | Paternal age (years) | | | | | | | |
| --- | --- | --- | --- | --- | --- | --- | --- | --- |
| **Stratification** | <25 | 25-29 | 30-34 | 35-39 | 40-44 | 45-49 | 50-54 | >54 |
| **Maternal Age** |  |  |  |  |  |  |  |  |
| Maternal age: <25 | 1.06 (0.43 to 2.63) | reference | 1.02 (0.37 to 2.80) | - | - | - |  | - |
| Maternal age: 25-34 | 1.52 (0.83 to 2.81) | reference | 1.02 (0.87 to 1.20) | 1.02 (0.81 to 1.30) | 1.05 (0.66 to 1.68) | 0.62 (0.20 to 1.95) |  | - |
| Maternal age: >34 | 0.61 (0.21 to 1.75) | reference | 0.50 (0.18 to 1.38) | 0.63 (0.22 to 1.77) | 0.63 (0.20 to 2.00) | - | - | 0.59 (0.06 to 5.37) |
| **Maternal BMI: kg/m^2^** |  |  |  |  |  |  |  |  |
| underweight (<18.5) | 1.25 (0.59 to 2.64) | reference | 1.00 (0.74 to 1.35) | 0.92 (0.59 to 1.46) | 1.34 (0.70 to 2.58) | 0.46 (0.06 to 3.43) | - | - |
| normal (18.5-24.9) | 1.07 (0.55 to 2.11) | reference | 1.04 (0.87 to 1.24) | 0.91 (0.71 to 1.18) | 0.88 (0.60 to 1.30) | 0.72 (0.37 to 1.42) | - | 0.79 (0.11 to 5.76) |
| overweight or obesity (>24.9) | 1.78 (0.23 to 13.97) | reference | 0.87 (0.42 to 1.79) | 1.39 (0.57 to 3.35) | 2.30 (0.73 to 7.31) | 5.21 (1.26 to 21.62) | - | - |
| **IVF** |  |  |  |  |  |  |  |  |
| Non-IVF | 1.21 (0.74 to 1.96) | reference | 1.00 (0.86 to 1.17) | 0.97 (0.78 to 1.21) | 1.05 (0.75 to 1.46) | 1.01 (0.57 to 1.78) | - | 0.76 (0.10 to 5.51) |
| IVF | - | reference | 1.32 (0.49 to 3.58) | 0.70 (0.22 to 2.19) | 0.88 (0.22 to 3.49) | 0.30 (0.03 to 3.14) | - | - |
| **Parity** |  |  |  |  |  |  |  |  |
| Nullipara | 1.37 (0.84 to 2.22) | reference | 1.05 (0.90 to 1.23) | 1.01 (0.80 to 1.28) | 0.96 (0.66 to 1.41) | 0.76 (0.39 to 1.48) | - | 0.74 (0.10 to 5.43) |
| Non-nullipara | - | reference | 0.78 (0.42 to 1.47) | 1.17 (0.60 to 2.31) | 1.61 (0.72 to 3.61) | 1.34 (0.42 to 4.25) | - | - |

Table S7 Sensitivity analysis for association between paternal age and LGA at 5 years interval

| **LGA: adjusted model, OR (95% CI)** | **Paternal age (years)** |  |  |  |  |  |  |  |
| --- | --- | --- | --- | --- | --- | --- | --- | --- |
| **Stratification** | <25 | 25-29 | 30-34 | 35-39 | 40-44 | 45-49 | 50-54 | >54 |
| **Maternal Age** |  |  |  |  |  |  |  |  |
| Maternal age: <25 | 1.00 (0.70 to 1.42) | reference | 1.20 (0.83 to 1.72) | 0.50 (0.18 to 1.41) | 1.87 (0.21 to 16.64) | - | - | - |
| Maternal age: 25-34 | 1.38 (1.05 to 1.81) | reference | 1.01 (0.95 to 1.01) | 1.01 (0.93 to 1.11) | 1.24 (1.05 to 1.47) | 1.36 (1.00 to 1.85) | 1.87 (1.04 to 3.35) | 1.83 (0.83 to 4.02) |
| Maternal age: >34 | 2.90 (0.45 to 18.76) | reference | 1.10 (0.71 to 1.70) | 1.13 (0.74 to 1.72) | 1.10 (0.72 to 1.68) | 1.01 (0.64 to 1.60) | 1.28 (0.73 to 2.24) | 1.05 (0.51 to 2.18) |
| **Maternal BMI: kg/m^2^** |  |  |  |  |  |  |  |  |
| underweight (<18.5) | 1.11 (0.64 to 1.92) | reference | 1.21 (1.00 to 1.45) | 1.20 (0.92 to 1.57) | 1.48 (1.00 to 2.19) | 1.04 (0.46 to 2.36) | 1.57 (0.46 to 5.39) | 1.96 (0.42 to 9.22) |
| normal (18.5-24.9) | 1.37 (1.09 to 1.73) | reference | 1.01 (0.95 to 1.07) | 1.08 (0.99 to 1.18) | 1.11 (0.98 to 1.25) | 1.12 (0.92 to 1.36) | 1.39 (0.97 to 1.99) | 1.05 (0.61 to 1.81) |
| overweight or obesity (>24.9) | 0.98 (0.50 to 1.92) | reference | 0.95 (0.81 to 1.11) | 0.89 (0.72 to 1.09) | 0.86 (0.64 to 1.16) | 0.63 (0.39 to 1.03) | 0.961 (0.36 to 2.54) | 2.23 (0.49 to 10.22) |
| **IVF** |  |  |  |  |  |  |  |  |
| Non-IVF | 1.29 (1.05 to 1.58) | reference | 1.00 (0.95 to 1.06) | 1.06 (0.98 to 1.15) | 1.11 (1.00 to 1.25) | 1.01 (0.84 to 1.22) | 1.23 (0.86 to 1.75) | 1.05 (0.61 to 1.82) |
| IVF | - | reference | 1.40 (0.99 to 1.97) | 1.27 (0.87 to 1.85) | 1.20 (0.75 to 1.90) | 1.55 (0.87 to 2.77) | 2.89 (1.23 to 6.81) | 2.79 (0.93 to 8.36) |
| **Parity** |  |  |  |  |  |  |  |  |
| Nullipara | 1.27 (1.02 to 1.56) | reference | 1.01 (0.95 to 1.07) | 0.99 (0.90 to 1.09) | 1.17 (1.00 to 1.36) | 1.13 (0.88 to 1.45) | 1.63 (1.04 to 2.55) | 1.46 (0.74 to 2.88) |
| Non-nullipara | 0.67 (0.30 to 1.52) | reference | 0.98 (0.86 to 1.12) | 0.97 (0.83 to 1.12) | 0.94 (0.78 to 1.13) | 0.94 (0.72 to 1.24) | 1.12 (0.70 to 1.80) | 0.98 (0.49 to 1.95) |
